# Supplementary material for: An ecological framework to understand the efficacy of fecal microbiota transplantation
Source: Nat Commun. 2020 Jul 3;11:3329. doi: 10.1038/s41467-020-17180-x (PMC7334230; doi:10.1038/s41467-020-17180-x)
Supplement: Supplementary file 1 — Supplementary Information [file 41467_2020_17180_MOESM1_ESM.pdf]

—SUPPLEMENTARY NOTES—
AN ECOLOGICAL FRAMEWORK TO UNDERSTAND THE EFFICACY OF FECAL MICROBIOTA TRANSPLANTATION

YANDONG XIAO[1,2], MARCO TULIO ANGULO[3,4], SONGYANG LAO[1], SCOTT T. WEISS[2], and YANG-YU LIU[2,5]

CONTENTS

1 Numerical Simulations of Fecal Microbiota Transplantation . . . 2
2 Network effect in microbial communities . . . 5
2.1 Net impacts . . . 5
2.2 Contribution matrix . . . 5
2.3 Comparing Net and direct impacts . . . 6
2.4 Graphical interpretation of the contribution matrix . . . 7
2.5 Previous studies on net impacts . . . 10
3 Algorithm for designing personalized probiotic cocktails . . . 10
3.1 Using the global network . . . 10
3.2 Using the ego network . . . 11
Supplementary References . . . 12

[1]COLLEGE OF SYSTEM ENGINEERING, NATIONAL UNIVERSITY OF DEFENSE TECHNOLOGY, CHANGSHA, HUNAN, 410073, CHINA, [2]CHANNING DIVISION OF NETWORK MEDICINE, BRIGHAM AND WOMEN'S HOSPITAL, AND HARVARD MEDICAL SCHOOL, BOSTON, MA 02115, USA, [3]INSTITUTE OF MATHEMATICS, UNIVERSIDAD NACIONAL AUTÓNOMA DE MÉXICO, JURIQUILLA 76230, MÉXICO, [4]NATIONAL COUNCIL FOR SCIENCE AND TECHNOLOGY (CONACYT), MEXICO CITY 03940, MÉXICO, [5]CENTER FOR CANCER SYSTEMS BIOLOGY, DANA-FARBER CANCER INSTITUTE, BOSTON MA 02115, USA

1. Numerical Simulations of Fecal Microbiota Transplantation

This section describes the details of FMT simulations presented in the main text. Consider a meta-community of N species following the Generalized Lotka-Volterra (GLV) model with an interaction matrix A and an intrinsic growth rate vector $\boldsymbol{r}$. Without loss of generality, we label species-1 as *C. difficile*.

Step 1. **Generate the interaction matrix A and the intrinsic growth rate vector $\boldsymbol{r}$.** The global ecological network $G(A)$ of the metacommunity can be constructed using a directed Erdős–Rényi random graph model with N nodes and connectivity C (i.e., there is a directed edge between any two ordered species with probability C). To generate the interaction matrix $A = [a_{ij}] \in \mathbb{R}^{N\times N}$ from this ecological network, for each edge $(j \to i) \in G(A)$ with $j \neq i$, we draw a_{ij} from a normal distribution $\mathcal{N}(0, \sigma^2)$ with $\sigma = 0.2$. All other entries of A are set to be zero. Additionally, we add a negative self loop $a_{ii} = -1$ to each node to ensure stability of the system. To generate the intrinsic growth rate vector $\boldsymbol{r} = [r_i] \in \mathbb{R}^N$, we draw each of its entries r_i from a uniform distribution $\mathcal{U}[0, 1]$. The parameters used to generate ecological networks for the simulations in Fig.3ef, Fig.4, Fig.5 and Fig.6cd of the main text are the following: $N = 100, C = 0.4, a_{ii} = -1, a_{ij} \sim \mathcal{N}(0, 0.2^2), r_i \sim \mathcal{U}[0, 1]$.

Step 2. **Generate healthy local communities corresponding to the gut microbiota of the donor and the initial healthy gut microbiota of the recipient**. We generate local communities by randomly selecting a subset of species from the metacommunity. To define a given local community as "healthy", we need to check if its species richness is sufficiently large and if it resists the invasion of *C. difficile*. To achieve that, we first make sure *C. difficile* is part of each local community. Then, we set the initial abundance profile $\boldsymbol{x}_0 \in \mathbb{R}^N$ by choosing $\boldsymbol{x}_{0,i} \sim \mathcal{U}[0, 1]$ if species i is present in the local community, and $\boldsymbol{x}_{0,i} = 0$ if it is not. As explained in the main text, to simulate the population dynamics of local communities we consider two cases:

a. Universal dynamics: local communities have the same GLV population dynamics as the metacommunity, characterized by the pair $(A, \boldsymbol{r})$. That is, the only difference between different local communities is the collection of species that are initially present.

b. Non-universal dynamics: each local community has its own population dynamics specified by a particular pair $(\tilde{A}, \tilde{\boldsymbol{r}})$. Here, we assume the growth rate vector $\tilde{\boldsymbol{r}}$ is different between subjects. Thus, for each subject $\tilde{r}_i$ is redrawn from $\mathcal{U}[0, 1]$. As explained in the main text, to specify $\tilde{A}$ for each local community we consider three sub-cases:

   * Host-Dependency-I. In this case the ecological networks and interaction types (i.e., the sign pattern) of $\tilde{A}$ and A are identical, but the interaction strengths are different. For example, $\tilde{a}_{ij} \neq a_{ij}$ but $\text{sgn}\,(\tilde{a}_{ij}) = \text{sgn}\,(a_{ij})$. To achieve that, for each non-zero a_{ij}, we draw $\tilde{a}_{ij}$ from the normal distribution $\mathcal{N}(0, \sigma^2)$. We then change the sign of $\tilde{a}_{ij}$, if it is different from the sign of a_{ij}.
   * Host-Dependency-II. In this case, the structure (i.e., the zero/nonzero pattern) of $\tilde{A}$ and A are identical, but the sign of the interactions and the interaction strengths may be different. To achieve that, for each non-zero a_{ij}, we just draw $\tilde{a}_{ij}$ from the normal distribution $\mathcal{N}(0, \sigma^2)$.
   * Host-Dependency-III. In this case the structure, interaction types and interaction strengths of $\tilde{A}$ and A can be all different. To achieve that, for each a_{ij}, we draw $\tilde{a}_{ij}$ from the normal distribution $\mathcal{N}(0, \sigma^2)$ with probability $p = 0.4$ and set it to be 0 otherwise.

After choosing the model parameters $(\tilde{A}, \tilde{\boldsymbol{r}})$ for each local community, we simulate its population dynamics using the initial condition $\boldsymbol{x}_0$ until the system reaches a steady state. If the steady-state abundance of *C. difficile* is less than 10^{-5} and the species richness is larger than a user-defined richness threshold, then we define the local community as "healthy". Otherwise,

it is discarded and Step 2 is repeated. For the results in the main text, we consider a meta-community of $N = 100$ species. Each healthy local community should contain at least 60 species.

Step 3. **Simulate the diseased state**. Starting from a healthy local community generated in Step 2 with its associated population dynamics $(\tilde{A}, \tilde{\boldsymbol{r}})$, we randomly remove part of its species (to mimic the impact of antibiotics administration). Then we simulate its population dynamics until the system reaches a steady state. If the steady-state abundance of *C. difficile* is larger than a user-defined diseased threshold, then the community is considered to be a "diseased state". In our simulations, we set the diseased threshold as 0.5.

Step 4. **Transplant the donor's microbiota to the recipient's diseased community**. To simulate the FMT process, we combine a diseased community with a healthy community by instantly changing the abundances of their common species and those donor-specific species. Then we simulate the population dynamics of the combined system (i.e., the post-FMT microbiota) following the recipient's microbial dynamics until the combined system reaches a new steady state. The efficacy of FMT is defined by a dimensionless variable, called the recovery degree, $\eta = \frac{x^{(d)} - x^{(p)}}{x^{(d)} - x^{(h)}}$, where x represents the abundance of *C. difficile*, and the superscripts (d), (p) and (h) represent the diseased, post-FMT, and initial healthy state, respectively.

Remark 1.

Note that in the FMT simulation, we don't have to forcibly set species abundance to zero based on any threshold value. In other words, we don't need to decide if a species will go to extinction asymptotically. This is because that any tiny residual species could recover to a high level of abundance after transplantation. For example, as shown in Supplementary Figure 1e (note that Supplementary Figure 1a-d are the same as Fig.2d-g in the main text), *C. difficile* and species-1 display exponential decay in the initial healthy microbiota. Interestingly, their abundances increase to very high levels after simulated antibiotics. Finally, after the simulated FMT, their abundances display exponential decay again. This result clearly implies that tiny residual species abundances do matter in our simulations.

Remark 2.

We emphasize that in the design of probiotic cocktails (see Supplementary Note 3), we do need to tell if a candidate cocktail will decolonize *C. difficile* or not. In other words, we need to know if *C. difficile* will go to extinction asymptotically. Mathematically, the structure of the ODEs in the GLV model does not allow for natural extinction in any finite time, i.e., that a species with initial abundance $x_i(0) > 0$ reaches the value $x_i(T) = 0$ for some finite time $T < \infty$. Note that the case of species eradication due to simulated antibiotic administration in our modeling framework certainly doesn't count as natural extinction. Therefore, during a natural evolution of the GLV model (without any simulated "antibiotics"), for any finite time $t > 0$, the species abundance $x_i(t)$ would never be exactly zero, unless it was absent at $t = 0$. This is because the coordinate planes ($x_i = 0$) are invariant manifolds for the GLV model, and since solutions of initial value problems for the GLV model are unique, it follows that natural extinction cannot occur in any finite time [1]. Any trajectory of the system with positive initial position remains in the first orthant for all time.

Supplementary Figure 2a,b showed that those initially present species might not co-exist in the long run. Mathematically, this means that a certain subset of initially present species will go to extinction asymptotically, i.e., their abundances vanish with time: $\lim_{t\to\infty} x_i(t) = 0$. Such asymptotic extinctions in the GLV model has been heavily studied before[1, 2, 3, 4, 5]. Those previous studies typically focused on special interaction types (either predator-prey or competitive) so that the interaction matrix A contains special sign patterns (either $\mathrm{sgn}(a_{ij}) = -\mathrm{sgn}(a_{ji})$ or $\mathrm{sgn}(a_{ij}) = \mathrm{sgn}(a_{ji}) < 0$ for all $i \neq j$), and then derived algebraic criteria on the model parameters $(\boldsymbol{r}, A)$, which guarantee that some species are driven to extinction asymptotically. Unfortunately, up to our knowledge, for general interaction types (or arbitrary sign patterns of the interaction matrix A), there are no analytical results to predict which species will be driven to extinction asymptotically.

Here, to better identify those species that will go to extinction asymptotically, we propose a novel approach, which is much better than arbitrarily choosing a threshold abundance.

(1) **Theoretical Preparation.**
We can prove that any asymptotical species extinction will end up with an exponential decay. Consider that species-i will go to extinction asymptotically, i.e., $\lim_{t\to\infty} x_i(t) = 0$, while all other species will approach their equilibrium abundance $x_j^* = x_j(\infty) > 0$. Note that species-i has dynamics:

$$\dot{x}_i = x_i(r_i + a_{ii}x_i + \sum_{j\neq i} a_{ij}x_j).$$

In the limit $t \to \infty$, we are close to $x_i(t) = 0$ and $x_j(t) = x_j^*$, we can get the first-order approximation for species-i's dynamics (by simply ignoring the second-order term $a_{ii}x_i^2$ and replacing $x_j(t)$ by x_j^*):

$$\dot{x}_i = x_i(r_i + \sum_{j\neq i} a_{ij}x_j^*) = -\lambda_i x_i,$$

where we have defined a constant $\lambda_i = -(r_i + \sum_{j\neq i} a_{ij}x_j^*)$. Then it is clear that $x_i(t)$ will decay exponentially, i.e., $x_i(t) \sim e^{-\lambda_i t}$ with $\lambda_i > 0$. The above argument can be easily extended to the case of multiple species going to extinction asymptotically. Using the same simulated data (as shown in Fig.2a of main text), but plotting the y-axis (species abundance) on the logarithmic scale, indeed we found that a few species' abundances display exponential decay, i.e., $x_i(t) \sim e^{-\lambda_i t}$ (see Supplementary Figure 2c), which is fundamentally different from the behavior of those co-existing species in the steady state.

(2) **Numerical procedure.**
First, to numerically distinguish exponential decay from steady-state behavior, we need to get each species' long-term abundance change rate λ_i, i.e., the slope of $x_i(t) \sim e^{-\lambda_i t}$ in the semi-log plot (see Supplementary Figure 2c,d), which can be obtained by fitting the asymptotical behavior of species abundances.

Second, to avoid introducing a threshold value of λ_i, we rank those species based on their λ_i values.

Third, we remove the top-K species one by one (based on the ranked λ_i values) from the system until we find the residual system has a feasible equilibrium (i.e., all the residual species have positive abundance in the steady state).

(i) In particular, each time we rearrange the species indices such that the $(N-K)$ residual species occupy the first $(N-K)$ entries, resulting in a reduced interaction matrix $A_{(K)} \in \mathbb{R}^{(N-K)\times(N-K)}$, and a reduced intrinsic growth vector $\boldsymbol{r}_{(K)} \in \mathbb{R}^{(N-K)\times 1}$.

(ii) We then calculate the equilibrium of the residual system, denoted as $\boldsymbol{x}^*_{(K)}$, by solving the linear equations:

$$\boldsymbol{x}^*_{(K)} = -A^{-1}_{(K)} \cdot \boldsymbol{r}_{(K)}.$$

If all the $(N-K)$ residual species have positive abundances at the equilibrium $\boldsymbol{x}^*_{(K)}$ (corresponding to their steady-state abundances in the infinite time limit), then we conclude that the top-K species will go to extinction asymptotically.

(3) **Demonstrations.**
Supplementary Figure 2e demonstrates the iteration process for the synthetic community shown in Supplementary Figure 2a. Note that the initial step $K = 0$ corresponds to the original system with all the $N = 15$ species present. When solving the linear equations for equilibrium, we find negative abundances (highlighted in blue), suggesting that the $N = 15$ species cannot co-exist in the steady state. After we remove the species with largest λ_i (i.e., species-7), the residual system still does not allow for a feasible equilibrium. Until we remove the top-5 species (i.e., species-7, 1, C, 15, 9), the resulting residual system permits a feasible

equilibrium (i.e., all the residual species have positive equilibrium abundances). We conclude that those top-5 species will go to extinction asymptotically.

Supplementary Figure 3 demonstrates the iteration process for a real microbial community associated with mouse experiments of antibiotic-mediated CDI (see [6] for experimental details). The interaction matrix has already been presented in main text Fig.7a. Supplementary Figure 3b showed that *Barnesiella*, und. Lachnospiraceae, and *Enterococcus* all display exponential decay in the long run of the simulation time window. Their fitted λ_i values are much larger than that of other taxa (Supplementary Figure 3c). However, the iterative process in Supplementary Figure 3d indicated that just excluding und. Lachnospiraceae and *Enterococcus* will already permit a feasible equilibrium for the residual system. Hence, we conclude that und. Lachnospiraceae and *Enterococcus* (rather than any more taxa) will go to extinction asymptotically. Note that the seemingly "large" decay rate of *Barnesiella* could be just due to the fact the simulation time is not long enough to capture the true asymptotical behavior of *Barnesiella*.

2. Network effect in microbial communities

In the main text, we discussed the intriguing network effect, i.e., those species that directly inhibit the growth of *C. difficile* might indirectly promote the growth of *C. difficile* through other "mediator" species. The net or effective impact of a species on the growth of *C. difficile* is hence largely context dependent. In this section, we systematically study the network effect in microbial communities. For simplicity, we focus on the GLV model.

2.1. **Net impacts.** Here we consider a metacommunity of N species labeled as $\Omega = \{1, \cdots, N\}$. We assume all local communities obtained from this metacommunity share universal population dynamics, hence different local communities just differ by their initial species collections.

Given a local community, denoted as ω, let us assume that its population dynamics is described by the GLV model

$$\frac{dx_i(t)}{dt} = x_i(t)[r_i^{(\omega)} + \sum_{j\in\omega} a_{ij}^{(\omega)} x_j(t)], \quad i \in \omega, \tag{1}$$

where $A^{(\omega)} = [a_{ij}^{(\omega)}] \in \mathbb{R}^{|\omega|\times|\omega|}$ and $\boldsymbol{r}^{\omega} \in \mathbb{R}^{|\omega|}$ are the interaction matrix and intrinsic growth rate vector of the local community ω, respectively. Here $|\omega|$ denotes the cardinality of the set ω. Note that the interaction matrix $A^{(\omega)}$ characterizes the direct ecological interactions (i.e., promotion, inhibition, or null) between any two microbial species of the local community. More precisely, species j has a direct promotion (inhibition or null) effect on species i's growth in the local community ω if and only if $a_{ij}^{(\omega)} > 0$ (< 0 or $= 0$, respectively).

Because species do not live in isolation but form a complex ecological system, the net effect of species j on species i depends not only on their direct interactions, but also on indirect interactions. For example, species j may directly inhibit species i and, at the same time, species j may directly promote species k that directly promotes species i. In this example, it is not trivial to conclude if the net effect of species j on species i is inhibition or promotion.

Consider two persisting species i and j (i.e., both species have non-zero steady-state abundances) in a local community ω. To quantify the net impact of species j on species i in this local community, we can calculate the contribution of species j on the steady-state abundance of species i, denoted as $s_{ij}^{(\omega)}$. Define the local contribution matrix $S^{(\omega)} \equiv [s_{ij}^{(\omega)}] \in \mathbb{R}^{|\omega|\times|\omega|}$. In the next subsection, we will analytically calculate the contribution matrix $S^{(\omega)}$ for the GLV model.

2.2. **Contribution matrix.** Here we derive the contribution matrix for a local community $\omega \subseteq \Omega$ assuming it reaches a steady-state abundance $\boldsymbol{x}^{*(\omega)}$. If some species in the community will go to extinction asymptotically, those species should be excluded from the subsequent analysis.

Under the two assumptions that the community follows GLV dynamics and permits a feasible equilibrium, the equilibrium of Supplementary Equation 1 is given by

$$\boldsymbol{x}^{*(\omega)} = -(A^{(\omega)})^{-1}\boldsymbol{r}^{(\omega)} = -\frac{\mathsf{adj}(A^{(\omega)})}{\mathsf{det}(A^{(\omega)})}\boldsymbol{r}^{(\omega)}, \tag{2}$$

where $\mathsf{adj}(A^{(\omega)}) \in \mathbb{R}^{n\times n}$ is a square matrix with dimension $n = |\omega|$, and its (i, j) entry is given by $[(-1)^{i+j}M_{ji}^{(\omega)}]$ and $M_{ji}^{(\omega)}$ is the (j, i)-minor of $A^{(\omega)}$, i.e., the determinant of the $(n-1)\times(n-1)$ submatrix by deleting j-th row and i-th column of $A^{(\omega)}$.

This equation implies that the steady-state abundance of species i is given by

$$x_i^{*(\omega)} = -\frac{1}{\mathsf{det}(A^{(\omega)})}\sum_{j\in\omega}(-1)^{i+j}M_{ji}^{(\omega)}r_j^{(\omega)}.$$

We define the contribution of species j on the steady-state abundance of species i in the local community ω as

$$s_{ij}^{(\omega)} := -\frac{(-1)^{i+j}M_{ji}^{(\omega)}r_j^{(\omega)}}{\mathsf{det}(A^{(\omega)})}. \tag{3}$$

Then naturally $x_i^{*(\omega)}$ is just the sum of the contributions from all species in the local community on species i, i.e.,

$$x_i^{*(\omega)} = \sum_{j\in\omega} s_{ij}^{(\omega)}.$$

In particular, species j has a net inhibition (promotion or null) effect on species i in the local community ω if and only if $s_{ij}^{(\omega)} < 0$ (> 0 or $= 0$, respectively). In what follows we use the shorthand $x_i^* = x_i^{*(\Omega)}$.

Remark 3. We recall the following facts from the contribution matrix.

a. Here we assume all species in the local community ω can persist because Supplementary Equation 3 only works for feasible communities, which require that $x_i^{*(\omega)} > 0$ for $i \in \omega$. For an infeasible community, we should exclude the extinct species from the global collection first, and form a new feasible local community. Then we can apply Supplementary Equation 3 for the new community to calculate the contribution matrix. Therefore, next subsection will introduce a numerical method to detect extinct species from an infeasible community.
b. For the classical GLV model, the functional response corresponds to Holling Type I $g_{LV}(x_i, x_j) = x_j$, which is linear in x_j. We note that the other common functional responses are nonlinear in x_j, such as Holling Type II ($g_{HII}(x_i, x_j) = \frac{c_1x_j}{1+c_1c_2x_j}$), DeAngelis-Beddington ($g_{DB}(x_i, x_j) = \frac{c_1x_j}{1+c_1c_2x_i+c_3x_j}$), and Crowley-Martin ($g_{CM}(x_i, x_j) = \frac{c_1x_j}{(1+c_1c_2x_i)(1+c_3x_j)}$). For these more complicated functional responses, quantifying the net effect of species j on the abundance of species i remains an open question.

2.3. **Comparing Net and direct impacts.** Due to complicated network effect, the net impact of species j on species i (denoted as $s_{ij}^{(\omega)}$) can be significantly different from the direct impact of species j on species i (denoted as $a_{ij}^{(\omega)}$). There are three cases:

(1) normal: direct and net impacts share the same sign, $\mathsf{sgn}(s_{ij}^{(\omega)}) = \mathsf{sgn}(a_{ij}^{(\omega)})$;
(2) bridging: direct impact is zero while net impact is not, $s_{ij}^{(\omega)} \neq 0$ but $a_{ij}^{(\omega)} = 0$;
(3) counter-intuitive: direct and net impacts have opposite signs, $\mathsf{sgn}(s_{ij}^{(\omega)}) = -\mathsf{sgn}(a_{ij}^{(\omega)}) \neq 0$.

In reality, two species that directly compete with (or benefit from) each other may effectively benefit from (or compete with) each other via interactions with the third species. These phenomena can be formalized as two special cases of the counter-intuitive case described above:

(3.1) $a_{ij} < 0, a_{ji} < 0$ but $s_{ij} > 0, s_{ji} > 0$;
(3.2) $a_{ij} > 0, a_{ji} > 0$ but $s_{ij} < 0, s_{ji} < 0$.

To illustrate the difference between direct and net impacts, we consider two examples:

Example 1. As an elementary example, consider a three-species metacommunity in Supplementary Figure 4a with species $\{C, 2, 3\}$. Consider that in the local community $\omega = \{C, 3\}$, where species 3 directly promotes species C, leading to a large abundance of species C. Note that in this metacommunity, species 2 directly inhibits species C (i.e., $a_{C2} < 0$), but it also directly promotes species 3 (i.e., $a_{32} > 0$) and hence indirectly promotes species C. It is not guaranteed that adding species 2 will decrease the abundance of species C. The net impact of species 2 on species C depends on the interaction strength a_{32}. If a_{32} is weak, the direct interaction dominates and adding species 2 will inhibit the growth of species C (blue curve in Supplementary Figure 4a). Otherwise, adding species 2 could increase the abundance of species C (red curve in Supplementary Figure 4a).

Example 2. Consider a more complicated metacommunity of 15 species shown in Supplementary Figure 4b. For this community, we calculated the contribution s_{Ci} of each species i on species C (see Supplementary Figure 4c). We found that many bridging cases (green dots below species index axis), as well as one counter-intuitive case (red triangle below species index axis). The counter-intuitive case exists because species 2 directly inhibits C (i.e., $a_{C2} < 0$), but its net effect is positive (i.e., $s_{C2} > 0$). Interestingly, calculating the contribution to species C using the subgraphs consisting of 1-step neighbors of species C, 2-step neighbors of species C, and 3-step neighbors of species C shows that the contribution values are dominated by the nearest neighbors (i.e., 1-step neighbors), see orange and green areas in Supplementary Figure 4b. This result suggests that for calculating the contribution matrix, instead of knowing the global network of the metacommunity, it is sufficient to know the local network consisting of nearest neighbors of the target species C. In network science, such a network is called the ego network of species C.

Here, to explicitly and systematically demonstrate the network effect using our ecological modeling framework based on ODEs, we performed extensive simulations to quantify the fractions of the three cases (i.e., normal, bridging, and counter-intuitive), as well as the two special cases (3.1) and (3.2), in synthetic ecological networks with GLV dynamics (see Supplementary Figure 5). In particular, we demonstrated the direct impact (encoded in the interaction matrix) in Supplementary Figure 5a, and the net impact (encoded in the contribution matrix) in Supplementary Figure 5b, for a community of $N = 15$ species. Note that in the contribution matrix, there are total 23 counter-intuitive cases (highlighted in red boxes), and 6 of those cases are special cases (3.1) and (3.2) (filled with stripe pattern). Supplementary Figure 5c systematically showed how the fractions of the three cases change with the community size N. Interestingly, the fractions remain quite stable over increasing community sizes. Even for a community of only $N = 10$ species, we already see the bridging and counter-intuitive effects. Supplementary Figure 5d demonstrated the fractions of the three cases with increasing connectance C of the microbial community. Here we find that the fractions change gradually over increasing connectance. In particular, denser networks (larger C) tend to have a higher fraction of counter-intuitive cases (shown in red) and a lower fraction of bridging cases (shown in green). Supplementary Figure 5e and f showed the fraction of the two special cases (3.1) and (3.2) with different N and C. We found that the two special cases are ubiquitous, especially in dense networks with large C.

2.4. **Graphical interpretation of the contribution matrix.** To offer graphical interpretation of the community matrix, let's consider a small community with 3 species.

Example 3. Consider a three-species community $\Omega = \{1, 2, 3\}$ that follows the GLV model with the following parameters:

$$A = \begin{pmatrix} a_{11} & 0 & 0 \\ a_{21} & a_{22} & 0 \\ 0 & a_{32} & a_{33} \end{pmatrix}, \quad \boldsymbol{r} = \begin{pmatrix} r_1 \\ r_2 \\ r_3 \end{pmatrix}.$$

We can directly calculate the steady state abundance of species 3 as

$$x_3^* = \frac{-r_1 a_{21} a_{32} + r_2 a_{11} a_{32} - r_3 a_{11} a_{22}}{a_{11} a_{22} a_{33}},$$

and note that $x_3^* = \sum_{i=1}^{3} s_{3i}$ if $x_3^* > 0$.

We also note that s_{3i} can be interpreted in terms of paths in the ecological network (Supplementary Table 1). For example, s_{31} contains $a_{21}a_{32}$ which is the product of the interactions in the path from node 1 to node 3. Note also that s_{ij} may contain interactions that are not in the direct path $(j \to i)$. For example, s_{32} contains the term a_{11}.

Contribution value of s_{3i} $(i = 1,2,3)$	M_{i3} $(i = 1,2,3)$	Graph interpretation of s_{3i}
$s_{31} = \frac{-M_{13} r_1}{\det(A)} = \frac{-r_1 a_{21} a_{32}}{\det(A)}$	$M_{13} = \begin{vmatrix} a_{11} & 0 & 0 \\ a_{21} & a_{22} & 0 \\ 0 & a_{32} & a_{33} \end{vmatrix} = a_{21}a_{32}$	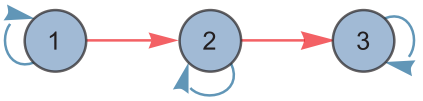
$s_{32} = \frac{M_{23} r_2}{\det(A)} = \frac{r_2 a_{11} a_{32}}{\det(A)}$	$M_{32} = \begin{vmatrix} a_{11} & 0 & 0 \\ a_{21} & a_{22} & 0 \\ 0 & a_{32} & a_{33} \end{vmatrix} = a_{11}a_{32}$	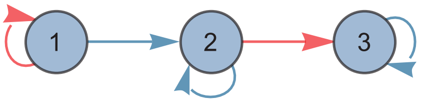
$s_{33} = \frac{-M_{33} r_3}{\det(A)} = \frac{-r_3 a_{11} a_{22}}{\det(A)}$	$M_{33} = \begin{vmatrix} a_{11} & 0 & 0 \\ a_{21} & a_{22} & 0 \\ 0 & a_{32} & a_{33} \end{vmatrix} = a_{11}a_{22}$	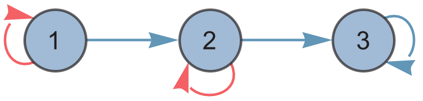

SUPPLEMENTARY TABLE 1. Contribution of other species to the steady-state abundance of species 3 in Example 3. The cross grain in the middle columns means the deletion of the i-th ($i = 1, 2, 3$) row and the 3rd column from the A matrix.

We next show that the observation in Example 3 is general in the sense that the net impact can be mapped into paths in the ecological network of a local community. To do this we first formally introduce the directed graph (i.e., the ecological network) associated with the interaction matrix A. In the analysis that follows, without loss of generality, we assume $\omega = \Omega$.

Definition 1. The directed graph $G(A)$**.** Given the interaction matrix $A = [a_{ij}] \in \mathbb{R}^{N\times N}$ we associate a directed graph $G(A) = (V, E)$ with vertex set V and edge set E. In this graph, nodes (or vertices) $V = \{x_1, \ldots, x_N\} := \{v_1, \ldots, v_N\}$ correspond to species, and the direct edges $E = \{(x_j \to x_i)|a_{ij} \neq 0\}$ correspond to direct ecological interactions (see Supplementary Figure 6a).

Definition 2. The bipartite graph $H(A)$**.** For our analysis, we will use the fact that any directed graph $G(A)$ can be represented a bipartite graph $H(A) = (V_A^+ \cup V_A^-, \Gamma)$. Here, $V_A^+ = \{x_1^+, \ldots, x_N^+\}$ and $V_A^- = \{x_1^-, \ldots, x_N^-\}$ are the set of vertices corresponding to the N rows and columns of the A matrix, respectively (See Supplementary Figure 6a). The edge set Γ is defined as $\Gamma = \{(x_i^+, x_j^-)|a_{ij} \neq 0\}$, which means that placing an edge (x_i^+, x_j^-) in the bipartite graph $H(A)$ if there is a directed edge $(x_j \to x_i)$ in the digraph $G(A)$ (i.e., there is a non-zero element a_{ij} in the A matrix). See Supplementary Figure 6b for an illustration.

Definition 3. The residual bipartite graph $H(\bar{A}_{ji})$**.** The key component in Supplementary Equation 3 is M_{ji}, which represents the determinant of the $(N-1)\times(N-1)$ submatrix formed by deleting the j-th row and i-th column from the A matrix. From the view of network science, this submatrix corresponds to a residual bipartite graph $H(\bar{A}_{ji})$ obtained by deleting the nodes x_j^+, x_i^- and their associated edges form the graph $H(A)$. More precisely, $H(\bar{A}_{ji}) = (V_A^+ - \{x_j^+\} \cup V_A^- - \{x_i^-\}, \Gamma')$ where Γ' is the subset of Γ by deleting the edges associated with nodes x_j^+ and x_i^- in Γ. Supplementary Figure 6c indicates $H(\bar{A}_{12})$ which is the key to quantify M_{12} in Supplementary Figure 6a.

Remark 4. From the above definitions, we know that the graphical interpretation of s_{ij} is hidden in the relations between M_{ji} and $H(\bar{A}_{ji})$. Thus, we recall the following facts from matrix theory [7].

a. **The determinant of a matrix**. The Leibniz formula for the determinant of an $N \times N$ matrix A is

$$\mathsf{det}(A) = \sum_{\sigma \in \Theta} \mathsf{sgn}(\sigma) \prod_{i=1}^{N} a_{i,\sigma_i}.$$

Above, Θ denotes the permutation group and $\mathsf{sgn}(\sigma)$ is the signature of the permutation σ. In matrix theory, the term $\prod_{i=1}^{N} a_{i,\sigma_i}$ is notation for the product of the entries at positions (i, σ_i), where i ranges from 1 to N. For example, the determinant of a 3×3 matrix A is

$$\begin{aligned}
&\sum_{\sigma \in \Theta} \mathsf{sgn}(\sigma) \prod_{i=1}^{N} a_{i,\sigma_i} \\
= \ &\mathsf{sgn}([1,2,3]) \prod_{i=1}^{N} a_{i,[1,2,3]_i} + \mathsf{sgn}([1,3,2]) \prod_{i=1}^{N} a_{i,[1,3,2]_i} + \mathsf{sgn}([2,1,3]) \prod_{i=1}^{N} a_{i,[2,1,3]_i} + \\
&\mathsf{sgn}([2,3,1]) \prod_{i=1}^{N} a_{i,[2,3,1]_i} + \mathsf{sgn}([3,1,2]) \prod_{i=1}^{N} a_{i,[3,1,2]_i} + \mathsf{sgn}([3,2,1]) \prod_{i=1}^{N} a_{i,[3,2,1]_i} \\
= \ &\prod_{i=1}^{N} a_{i,[1,2,3]_i} - \prod_{i=1}^{N} a_{i,[1,3,2]_i} - \prod_{i=1}^{N} a_{i,[2,1,3]_i} + \prod_{i=1}^{N} a_{i,[2,3,1]_i} + \prod_{i=1}^{N} a_{i,[3,1,2]_i} - \prod_{i=1}^{N} a_{i,[3,2,1]_i} \\
= \ &a_{1,1}a_{2,2}a_{3,3} - a_{1,1}a_{2,3}a_{3,2} - a_{1,2}a_{2,1}a_{3,3} + a_{1,2}a_{2,3}a_{3,1} + a_{1,3}a_{2,1}a_{3,2} - a_{1,3}a_{2,2}a_{3,1}.
\end{aligned}$$

b. **The determinant of a matrix associates with all the perfect matchings of its corresponding bipartite graph**. For a given A matrix, $\prod_{i=1}^{N} a_{i,\sigma_i}$ for $\sigma \in \Theta$ corresponds to the product of one perfect matching of its corresponding bipartite graph $H(A)$. Thus, $\sum_{\sigma \in \Theta} \prod_{i=1}^{N} a_{i,\sigma_i}$ consists of all the perfect matchings of $H(A)$.

In summary, we can make the following observations:

Remark 5.

a. The determinant $\mathsf{det}(A)$ equals the sum of the weights of all perfect matchings multiplied by their respective signatures. Thus, the perfect matchings reflect the relation between a_{ij} and $\mathsf{det}(A)$ from a graph viewpoint.
b. M_{ji}, the (j,i)-minor of matrix A, is the determinant of submatrix by deleting j-th row and i-th column from the A matrix, which associates with all the perfect matchings of the residual bipartite graph $H(\bar{A}_{ji})$.
c. The contribution value s_{ij} associates with all the perfect matchings of the residual bipartite graph $H(\bar{A}_{ji})$.

Consider the microbial community of Supplementary Figure 7a. Here, the net impact of species 2 on species 1 is given by

$$s_{12} = -\frac{(-1)^{(1+2)} M_{21} r_2}{\mathsf{det}(A)} = \frac{a_{32}a_{43}a_{14}a_{55}r_2}{\mathsf{det}(A)}.$$

The numerator $M_{21} = a_{32}a_{43}a_{14}a_{55}$ consists of the product of $(N-1)$ direct interactions corresponding to the perfect matchings of $H(\bar{A}_{21})$ (see Supplementary Figure 7b). Each perfect matching in $H(\bar{A}_{21})$ has two parts: the direct interactions in the path from species 2 to species 1 (green in Supplementary Figure 7b), and the "supplementary" elements which are direct interactions disjoint to such path (orange in Supplementary Figure 7b). Thus, these supplementary elements contain direct interactions that are not part of the paths connecting species 2 and species 1.

Supplementary Figure 7c highlights the perfect matching of M_{21} in the original ecological network using green edges and nodes (indicating the path from species 2 to 1) and the orange edge and node (representing the supplementary element). Supplementary Figures 7d-f show more complicated examples. Here, due to the three perfect matchings in Supplementary Figure 7e, the expression for M_{21}

consists of three terms, as shown in Supplementary Figure 7f, and each term corresponds to a path from species 2 to 1 and their supplementary elements.

The above discussion shows that s_{ij} depends on both "direct" paths from j to i, and "supplementary elements" that are not in those paths. The presence of supplementary elements is hard to predict using a purely ecological reasoning, and they can only be revealed by a mathematical analysis.

2.5. **Previous studies on net impacts.** Several previous studies have analyzed the response of ecosystems to the so-called "press perturbations" [8, 9, 10, 11]. These press perturbations capture how the abundance of species i will change in response to a change in the abundance of species j. For example, in [10], the authors introduce the "net effect matrix" $K = -A^{-1}$ as follows:

> "*Each element of* $-A^{-1}$ *specifies the direction and magnitude by which the abundance of the species in row* i *is expected to respond to a perturbation of the species in column* j*.*"

The net effect matrix and our contribution matrix have the same aim, that is, quantifying the impact of one species on the abundance of another species. However, we emphasize three important differences:

Remark 6.

a. The net effect matrix K quantifies the effect of an infinitesimal change in the abundance of one species to other species in the community [10]. Namely, this matrix captures the change in abundance during the process to reach new equilibrium after adding a small "press" perturbation.
b. The contribution matrix S assesses the net impact of the presence of one species on the steady-state abundance of another species. That is, we quantify the effects on the new steady-state abundance after adding new species (e.g., due to a transplantation). Crucially, adding some species do not necessarily result in a small perturbation to the steady-state abundance. Because of this fact, the contribution matrix is more suitable for analyzing and designing FMT than the net effect matrix.
c. The contribution matrix actually involves the growth rate vector $\boldsymbol{r}$, while the net effect matrix K is independent of $\boldsymbol{r}$.
d. The sign of K equals to the sign of S, but the magnitude of each entry is in general different. That is, $\mathsf{sgn}(S) = \mathsf{sgn}(K)$ but, in general, $s_{ij} \neq k_{ij}$.

3. Algorithm for designing personalized probiotic cocktails

3.1. **Using the global network.** Given a metacommunity of N species and its global ecological network $G(A)$. Our aim is to identify a subset of species (called a probiotic cocktail) to effectively promote or inhibit the growth of a target species in any "diseased" local community, where the target species has abnormally low or high abundance. Here we describe the algorithm in the particular case of inhibiting one species, i.e., *C. difficile*, but it can be easily generalized to inhibit or promote any other species.

Step 1. Calculate the contribution matrix S from the interaction matrix A of the metacommunity, quantifying the net impact between any two species.

Step 2. Consider both direct $(a_{Cj} < 0)$ and effective $(s_{Cj} < 0)$ inhibitors as the "global inhibitors" from the metacommunity. Let the initial cocktail contain all those global inhibitors that are not present in the diseased local community (i.e., the patient's disrupted microbiota).

Step 3. If transplanting the initial cocktail to the patient microbiota will decolonize *C. difficile*, the procedure terminates. If not, go to Step 4.

Step 4. Calculate the local contribution matrix using the new local community consisting of all species in the patient's diseased microbiota and all species in the initial cocktail. For each species in the initial cocktail, we numerically test if it is an effective inhibitor (i.e., has a negative net impact on the growth of *C. difficile*) in the restored local community. The species is kept in

the cocktail if it is an effective inhibitor, and it is removed from the cocktail if it is not an effective inhibitor.

Step 5. Repeat Step 4 until all the species in the cocktail are effective inhibitors in the local community.

Step 6. Return the final cocktail as the personalized probiotic cocktail.

To better explain the workflow of our algorithm, we presented a schematic diagram of the algorithm, shown in Supplementary Figure 8. Some remarks are in order:

Remark 7.

a. At the beginning, the algorithm assembles an initial cocktail with all global inhibitors. Thus, the final personalized patient-specific cocktail is a subset of those global inhibitors.
b. Note that, in a community of N species, the complexity of a brute-force algorithm that test if each possible species is an inhibitor is of the order $\mathcal{O}(2^{N-1})$. The contribution matrix actually avoids this "curse of dimensionality", allowing us to search only in a reduced pool of candidates given by the initial cocktail of global inhibitors.
c. Note that, because of the network effects, an species may have different net impacts in different local communities simply because the species collections are different. Therefore, it is necessary to check at each step that the possible candidates play the desired net impact in the local microbial community.
d. Note that for a local community that doesn't permit a feasible equilibrium, we first need to exclude those species that are going to extinction asymptotically (which can be identified via the approach discussed in Remark 2 of Supplementary Note 1). We end up with a new local community with a feasible equilibrium. Then we calculate the contribution matrix of the new community. Of course, those extinct species will not contribute to the equilibrium of the new community.

3.2. **Using the ego network.** In Supplementary Figure 4c, we showed how the net impacts are dominated by the subnetwork consisting of all species that are nearest neighbors of the target species. This result suggests that just knowing the ego network of the target species is enough to calculate the net impacts, instead of knowing the global ecological network.

The ego network of *C. difficile* consists of a focal node/species ("ego", i.e., *C. difficile*), those nodes/species to which *C. difficile* directly interacts with (they are called "alters"), the links/interactions between *C. difficile* and its alters, as well as the links/interactions among the alters (see Supplementary Figure 4b). The algorithm to suppress *C. difficile* using its ego network is almost same with the previous algorithm using the global ecological network. The only difference is the first-step, where we just use the ego network to calculate the contribution matrix. The rest steps are the same.

Some remarks are in order:

Remark 8.

a. In the above algorithm, we only use the information of the ego network of *C. difficile*.
b. The cocktails are personalized because different patients have different remaining species in their diseased microbiota, and thus we will have different ego networks of *C. difficile*.
c. Our algorithm can be generalized to consider a k-step ego network instead of the 1-step ego network used above. Using an ego network with slightly larger step may significantly improve the efficacy of the designed cocktail. For example, as shown in Supplementary Figure 9, by using the 1-step ego network, our algorithm yields the cocktail $R_{\text{ego-1}} = \{10\}$. The performance of this cocktail is worse than that of the cocktail R_{global} designed using the global ecological network (see dashed line in Supplementary Figure 9). However, if we consider the 2-step ego network, our algorithm yields the cocktail $R_{\text{ego-2}} = \{5, 10, 12, 13\}$ whose performance is almost identical to that of R_{global} (Supplementary Figure 9).

Supplementary References

[1] Hallam, T. G., Svoboda, L. J. & Gard, T. C. Persistence and extinction in three species lotka-volterra competitive systems. *Mathematical Biosciences* **46**, 117–124 (1979).

[2] Ahmad, S. & Lazer, A. C. One species extinction in an autonomous competition model. In *World Congress of Nonlinear Analysis*, vol. 92, 359–368 (1996).

[3] Zeeman, M. L. Extinction in competitive lotka-volterra systems. *Proceedings of the American Mathematical Society* **123**, 87–96 (1995).

[4] Deoca, F. M. & Zeeman, M. Balancing survival and extinction in nonautonomous competitive lotka-volterra systems. *Journal of mathematical analysis and applications* **192**, 360–370 (1995).

[5] Ackleh, A. S., Marshall, D. F. & Heatherly, H. E. Extinction in a generalized lotka-volterra predator-prey model. *International Journal of Stochastic Analysis* **13**, 287–297 (1900).

[6] Stein, R. R. *et al.* Ecological modeling from time-series inference: insight into dynamics and stability of intestinal microbiota. *PLoS Comput Biol* **9**, e1003388 (2013).

[7] Trefethen, L. N. & Bau III, D. *Numerical linear algebra*, vol. 50 (Siam, 2013).

[8] Bender, E. A., Case, T. J. & Gilpin, M. E. Perturbation experiments in community ecology: theory and practice. *Ecology* **65**, 1–13 (1984).

[9] Montoya, J., Woodward, G., Emmerson, M. C. & Solé, R. V. Press perturbations and indirect effects in real food webs. *Ecology* **90**, 2426–2433 (2009).

[10] Novak, M. *et al.* Characterizing species interactions to understand press perturbations: what is the community matrix? *Annual Review of Ecology, Evolution, and Systematics* **47**, 409–432 (2016).

[11] Giordano, G. & Altafini, C. Qualitative and quantitative responses to press perturbations in ecological networks. *Scientific Reports* **7**, 11378 (2017).

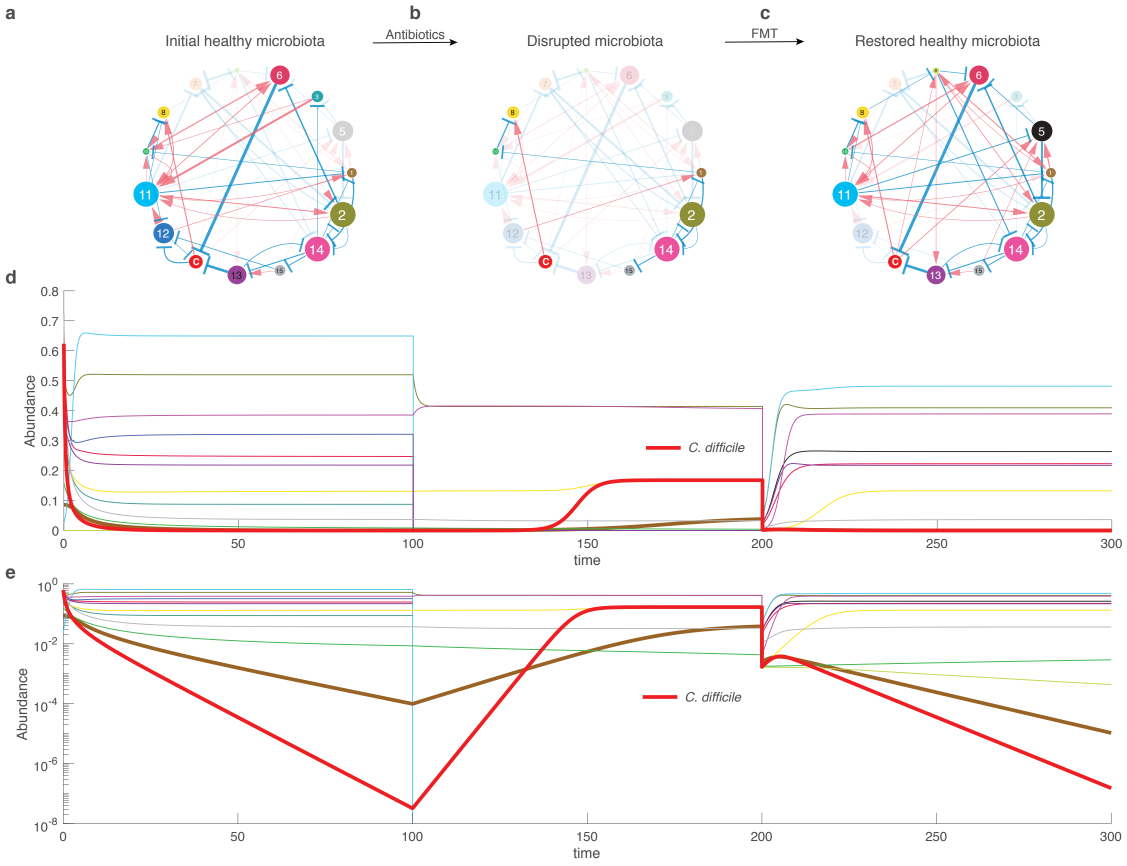

SUPPLEMENTARY FIGURE 1. **Temporal behavior of species abundances in the simulated FMT process.** We start from an initially "healthy" community (**a**), simulate the impact of antibiotic administration by eradicating some species from the community (**b**), restore the healthy community by transplanting species from another healthy community (**c**). The simulation details of the FMT process is the same as shown in Fig.2d-g of the main text. The simulated time series of species abundances with linear (**d**) or logarithmic scale (**e**).

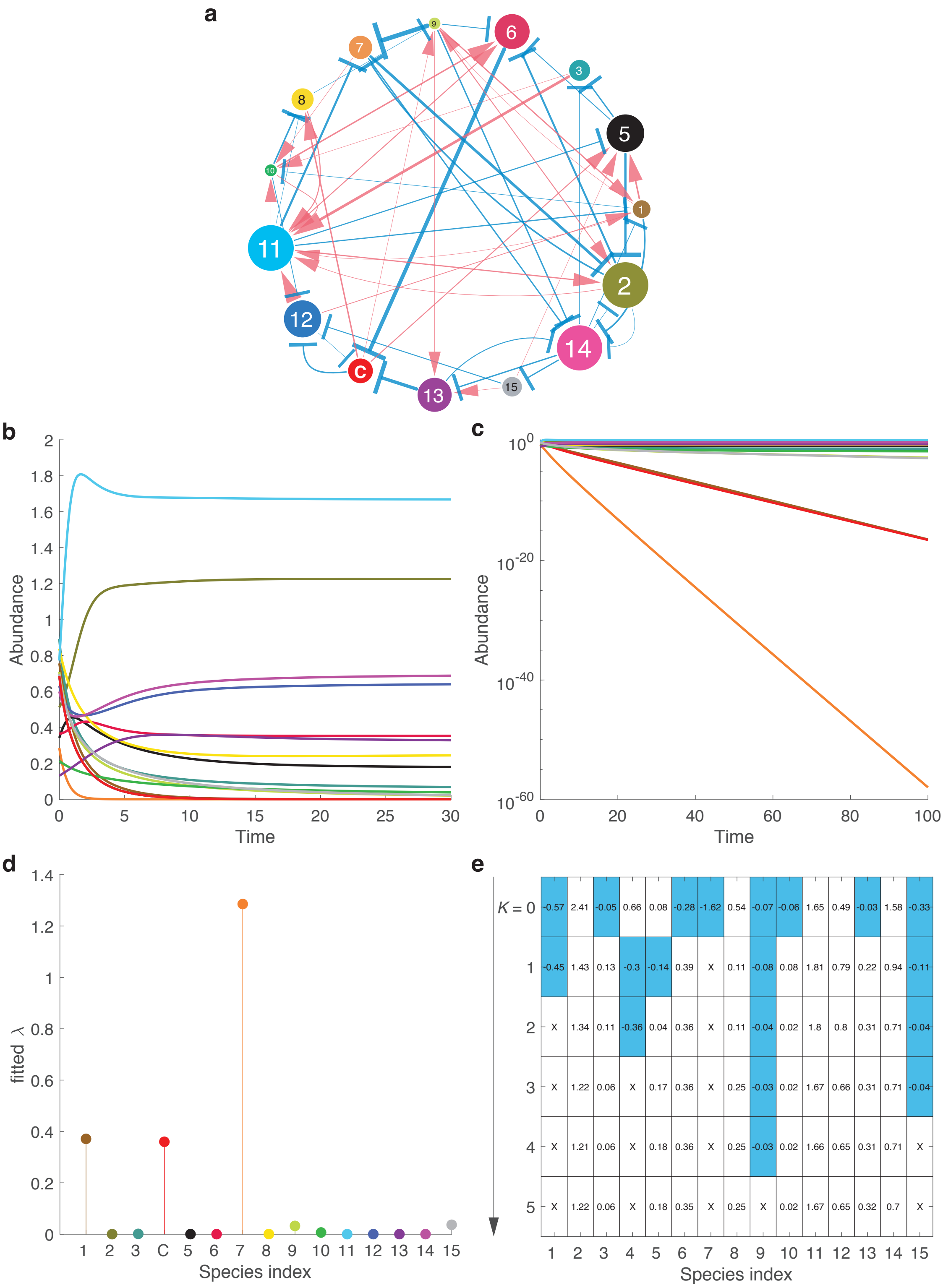
a
9
6
7
3
8
5
10
1
11
2
12
14
c
13
15
b
Abundance
Time
c
Abundance
Time
d
fitted λ
Species index
1 2 3 C 5 6 7 8 9 10 11 12 13 14 15
e
K = 0
-0.57 2.41 -0.05 0.66 0.08 -0.28 -1.62 0.54 -0.07 -0.06 1.65 0.49 -0.03 1.58 -0.33
1
-0.45 1.43 0.13 -0.3 -0.14 0.39 X 0.11 -0.08 0.08 1.81 0.79 0.22 0.94 -0.11
2
X 1.34 0.11 -0.36 0.04 0.36 X 0.11 -0.04 0.02 1.8 0.8 0.31 0.71 -0.04
3
X 1.22 0.06 X 0.17 0.36 X 0.25 -0.03 0.02 1.67 0.66 0.31 0.71 -0.04
4
X 1.21 0.06 X 0.18 0.36 X 0.25 -0.03 0.02 1.66 0.65 0.31 0.71 X
5
X 1.22 0.06 X 0.18 0.35 X 0.25 X 0.02 1.67 0.65 0.32 0.7 X
1 2 3 4 5 6 7 8 9 10 11 12 13 14 15
Species index

SUPPLEMENTARY FIGURE 2. **Asymptotic extinction in the generalized Lotka-Volterra model. a**. The ecological network of a microbial community with 15 species. The ecological network is the same as shown in Fig.2a of the main text. Blue (or red) edges represent the inhibition (or promotion) impacts between species. **b**. Starting from an arbitrary initial condition, we can numerically solve the ODEs and obtain the time series of species abundances. **c**. Plotting the species abundances on the logarithmic scale demonstrates that some species' abundances decay exponentially in the long run, i.e., $x_i(t) \sim e^{-\lambda_i t}$ for large t. **d**. The decay rate λ_i obtained by fitting the asymptotic behavior of each species' abundance time series. We notice that species-1,C, 7, 9, and 15 display noticeable decay rate, suggesting that they will go to extinction asymptotically. **e**. To avoid choosing a subjective threshold value of λ_i to identify those species that will go to extinction asymptotically, we develop a heuristic method. In particular, we rank those species based on their λ_i values. Then we remove the top-K species one by one from the system until we find the residual system permits a feasible equilibrium (i.e., all the residual species have positive abundances in equilibrium). Here, the K-th row represents the equilibrium abundance profile calculated by solving the linear equation $\boldsymbol{x}^*_{(K)} = -A^{-1}_{(K)} \cdot \boldsymbol{r}_{(K)}$ for the residual system. Removed species are highlighted by 'X', residual species with negative equilibrium abundances are highlighted in blue.

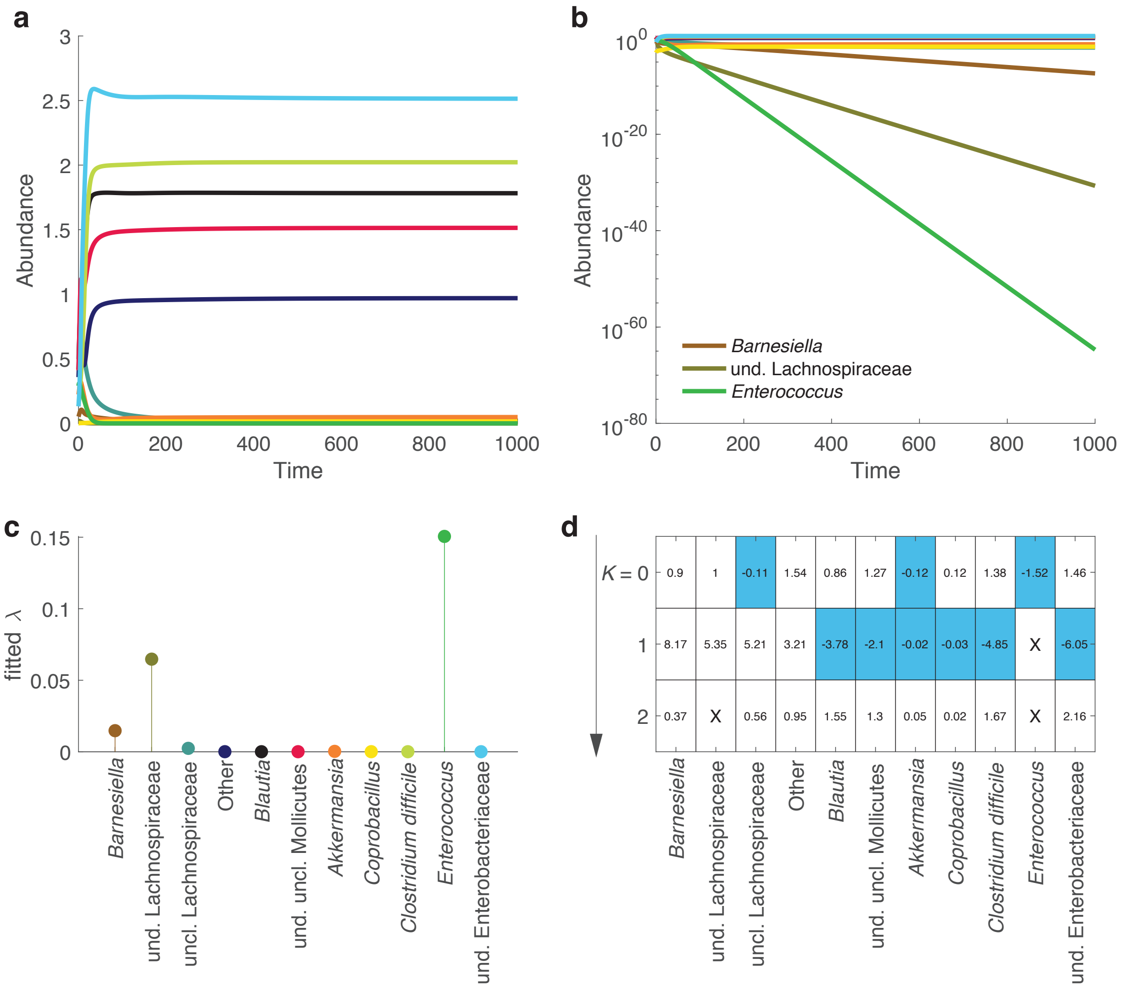

SUPPLEMENTARY FIGURE 3. **Asymptotic extinction in a real microbial community.** The interaction matrix (inferred from the mouse experiments of antibiotic-mediated CDI, see Ref.[69] of the main text) was presented in Fig.7a of the main text. **a**. Starting from an arbitrary initial condition, we can numerically solve the ODEs and obtain the time series of taxa abundances. **b**. Plotting the taxa abundances on the logarithmic scale demonstrates that the abundances of *Barnesiella*, und. Lachnospiraceae, *Enterococcus* decay exponentially, i.e., $x_i(t) \sim e^{-\lambda_i t}$ for large t. **c**. The decay rate λ_i obtained by fitting the asymptotic behavior of each species' abundance time series. *Barnesiella*, und. Lachnospiraceae, *Enterococcus* display noticeable non-zero decay rate. **d**. The iterative process showed that excluding und. Lachnospiraceae and *Enterococcus* can already permit the residual system to have a feasible equilibrium. This suggests that und. Lachnospiraceae and *Enterococcus* will go to extinction asymptotically.

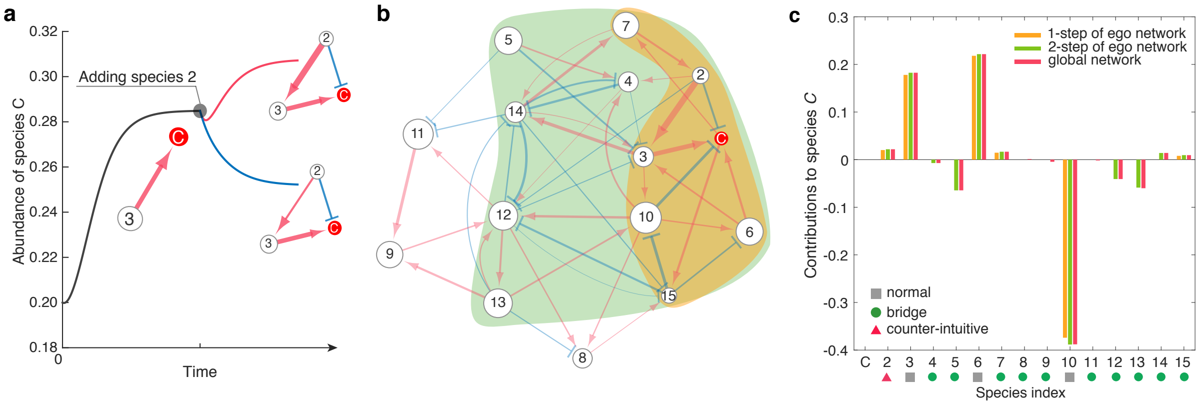

SUPPLEMENTARY FIGURE 4. **Network effect. a.** Two ecological networks with the same structure and sign-patterns, but different interaction strengths lead to different net impacts of species 2 on species C. **b.** The ecological network of a metacommunity with 15 species. We highlight the 1st-step and 2nd-step ego-networks of the target species C in orange and green, respectively. **c.** The contributions of other species to species C's steady-state abundance calculated from the 1st-step and 2nd-step ego-networks of C and the global ecological network, respectively.

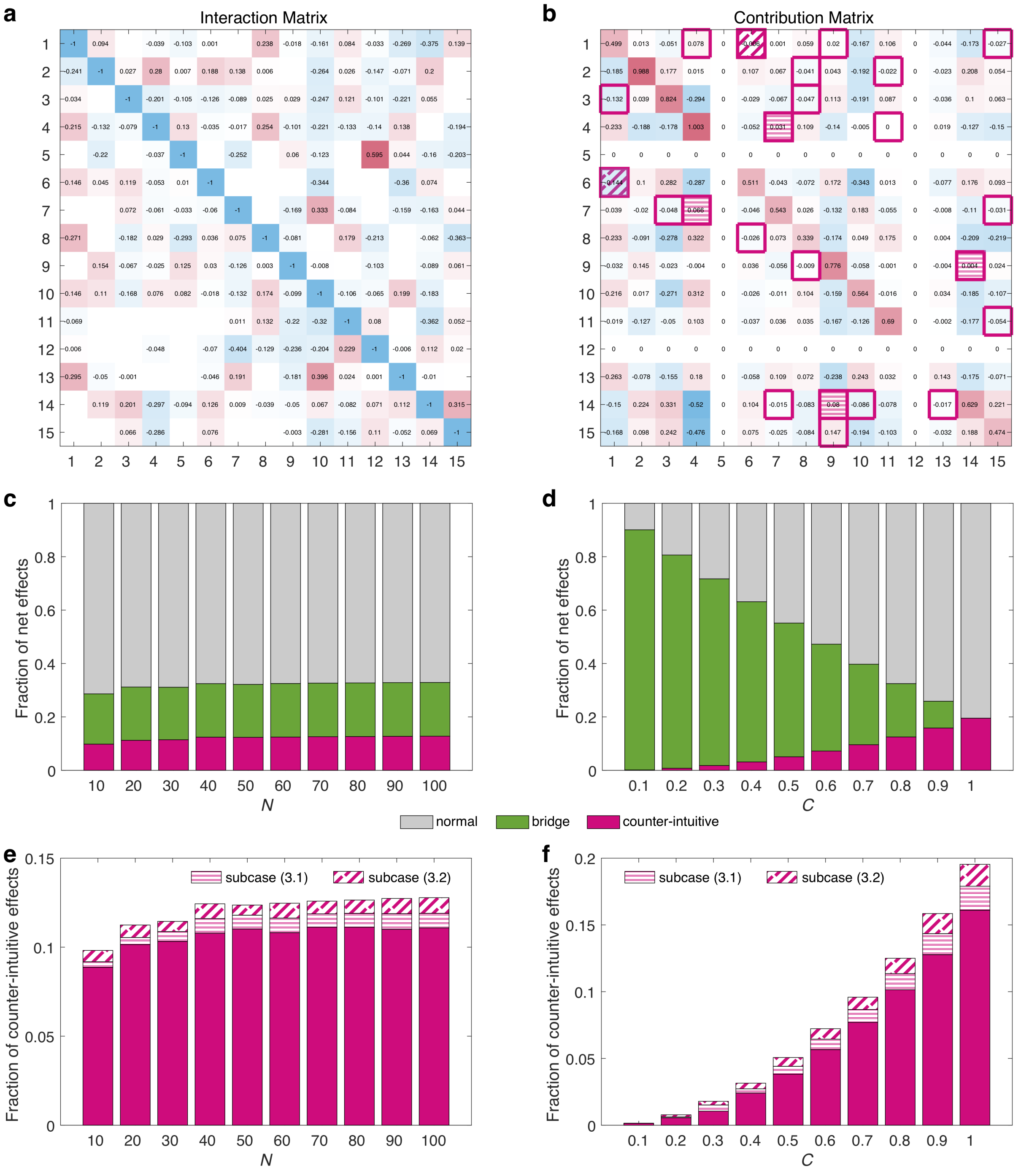

SUPPLEMENTARY FIGURE 5. **The ubiquity of network effect in the GLV model.** **a**. The interaction matrix of a community with 15 species governed by GLV dynamics. **b**. The contribution matrix of the community in a. Red boxes highlighted the counter-intuitive cases, while red boxes with stripe patterns indicated the two subcases (3.1) and (3.2). **c**. Fractions of the three main cases of network effect as functions of the community size. We set the intra-species interaction $a_{ii} = -1$, the inter-species interaction $a_{ij} \sim \mathcal{N}(0, 0.2^2)$, the connectance of ecological network $C = 0.8$ (the probability that species-i interacts with species-j), the growth rate of each species $r_i \sim \mathcal{U}[0, 1]$. For each N, we ran 20 different realizations. **d**. Fractions of the three main cases of network effect as functions of the network connectance. We set $N = 100$, $a_{ii} = -1$, $a_{ij} \sim \mathcal{N}(0, 0.2^2)$, $r_i \sim \mathcal{U}[0, 1]$. For each C, we ran 20 different realizations. **e,f** Fraction of counter-intuitive cases of network effect as a function of community size (N) or network connectance (C). Here, each bar represents the total fraction of counter-intuitive cases, and the parts with filled-in stripe patterns indicate the fractions of the two special cases (3.1) and (3.2).

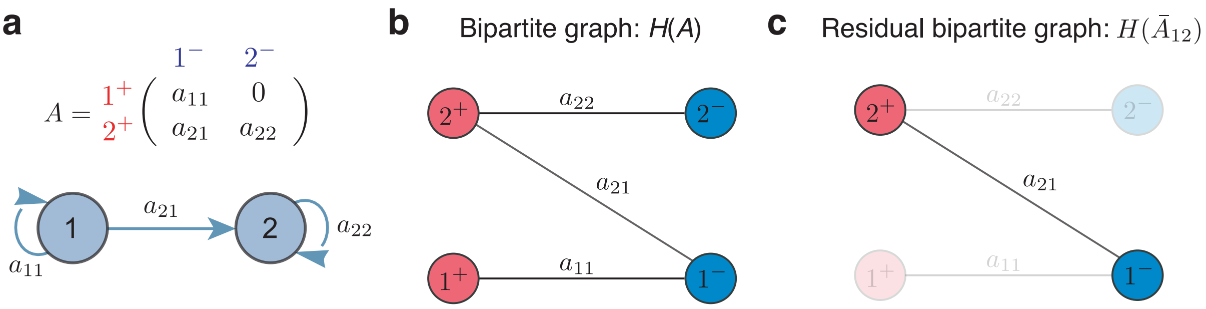

Supplementary Figure 6. A **matrix and its bipartite representation. a.** An illustration of how an A matrix can be represented by a directed graph with N nodes. **b.** The corresponding bipartite graph $H(A)$ of the A matrix in panel a. **c.** The residual bipartite graph $H(\bar{A}_{12})$ corresponds to the submatrix by deleting the first row and second column (the light nodes and edges) from the A matrix in panel a.

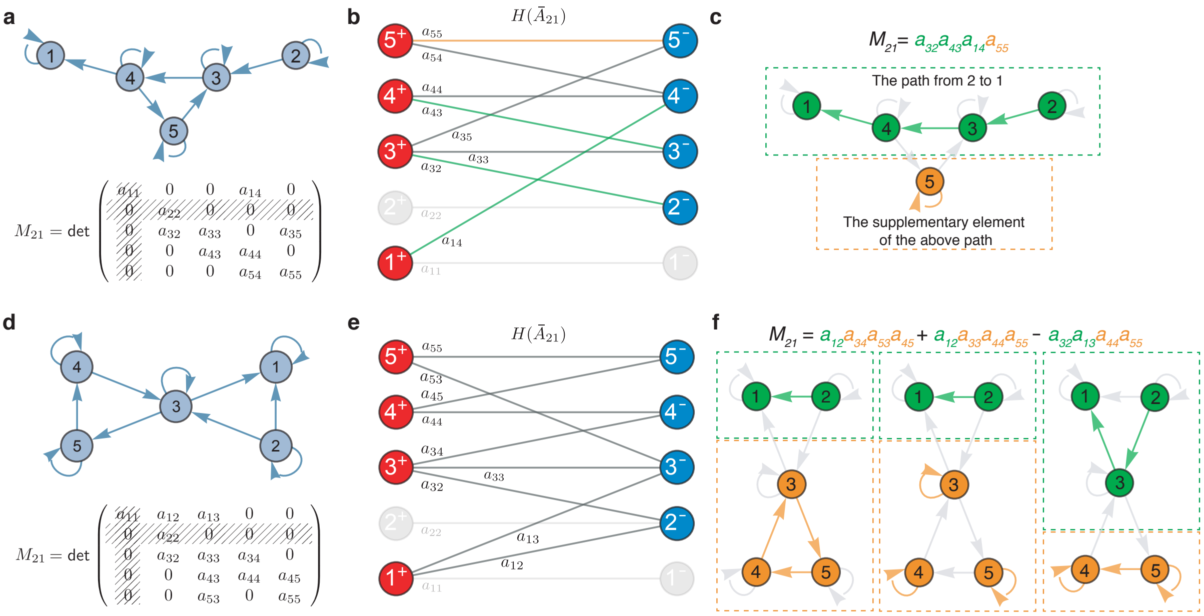

SUPPLEMENTARY FIGURE 7. **The graph interpretation of net impacts. a.** The ecological network of a metacommunity of 5 species. Here the term M_{21} involved in s_{12} quantifies the net effect of species 2 on species 1. **b.** The bipartite graph of the A matrix in panel a. Note that $H(\bar{A}_{21})$ corresponds to a residual bipartite graph by deleting the 2nd row and 1st column in the A matrix. The light gray nodes and links are deletions. The orange and green links represent the perfect matching of $H(\bar{A}_{21})$. **c.** M_{21} is composed of the perfect matching of $H(\bar{A}_{21})$, which has two disjoint parts: (i) the path from species 2 to species 1 (shown in green); and (ii) the supplementary elements of this path (shown in orange). **d.** M_{21} in a metacommunity with a more complicated network. **e.** There exists three perfect matchings in this residual bipartite graph, i.e, $a_{12}a_{34}a_{53}a_{45}, a_{12}a_{33}a_{44}a_{55}$ and $a_{32}a_{13}a_{44}a_{55}$. **f.** M_{21} is composed of three perfect matchings of $H(\bar{A}_{21})$. Similar with panel c, we highlight the three paths from species 2 to species 1 (shown in green) and their corresponding supplementary elements (shown in orange), respectively.

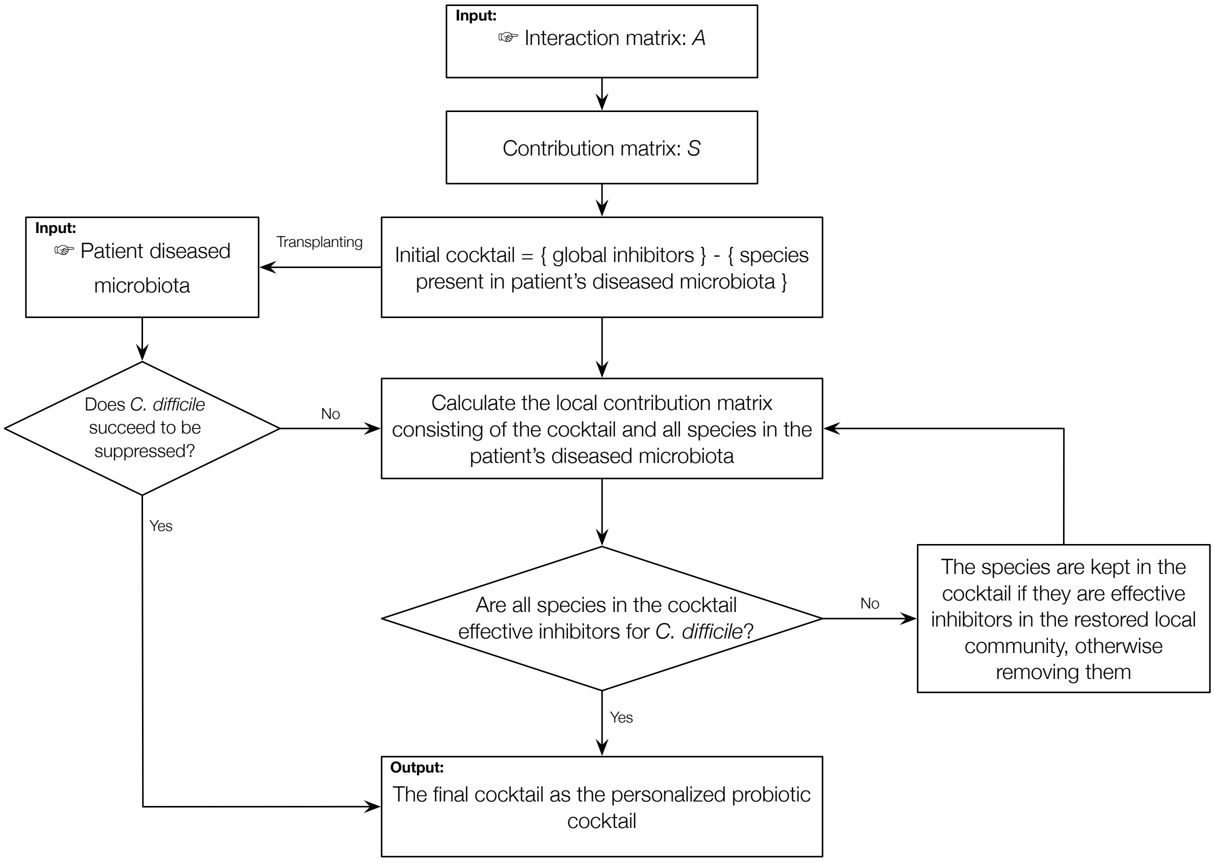

SUPPLEMENTARY FIGURE 8. **The workflow of our algorithm for the design of personalized probiotic cocktail.**

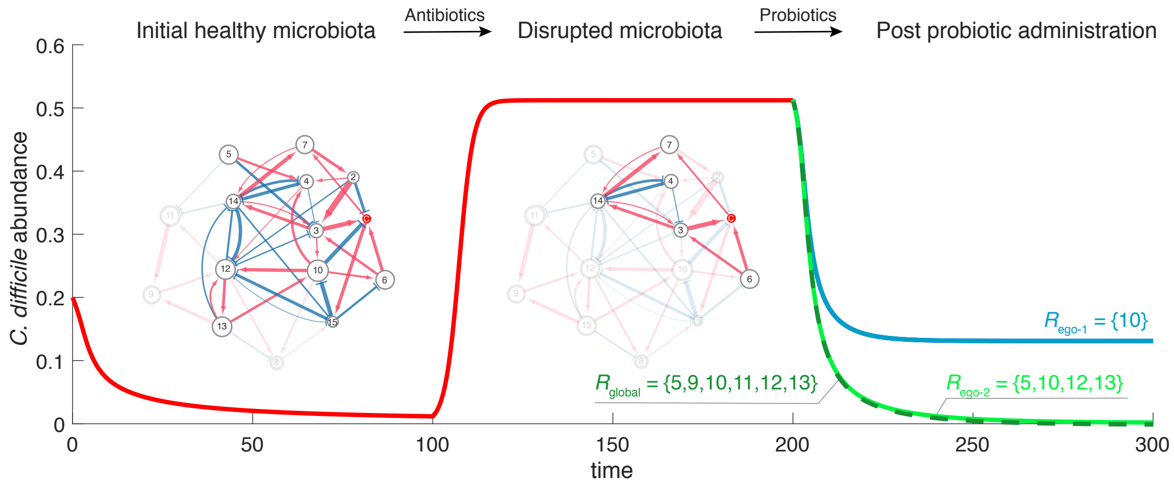

SUPPLEMENTARY FIGURE 9. **Rationally design probiotic cocktails to decolonize *C. difficile*.** The trajectories show the abundance of *C. difficile* in an initially healthy microbiota, a disrupted microbiota, and the restored microbiota with probiotic cocktails designed using the global ecological network, 1-step and 2-step ego networks of *C. difficile*, i.e., R_{global}, $R_{\text{ego-1}}$ and $R_{\text{ego-2}}$, respectively.

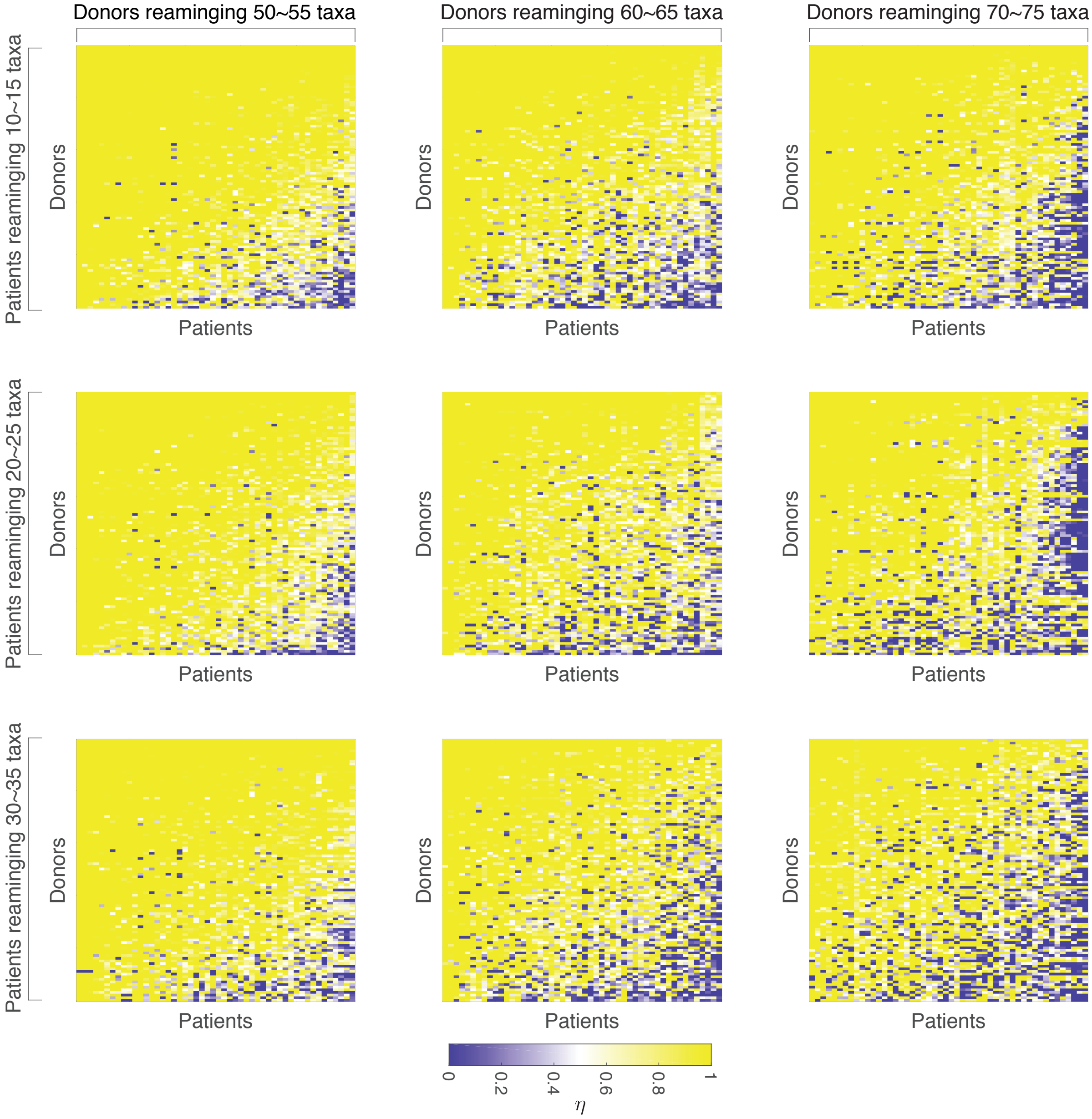

SUPPLEMENTARY FIGURE 10. **Donor-recipient compatibility issue matters.** We simulate the FMT process for each of the 100×50 (donor, recipient) pairs, and plot the recovery degree in a 100×50 matrix, where rows (or columns) are sorted based on the average recovery degree of each row (or column). The resulting healthy microbiota of those 100 donors contains 50∼55 (left column), 60∼65 (middle column), and 70∼75 (right column) species. From top to bottom, the pre-FMT microbiota of 50 recipients contain 10∼15 (top), 20∼25 (middle), and 30∼35 (bottom) species, respectively. In simulations, the underlying ecological network is generated from a directed random graph model with connectivity 0.4, and the ecological interactions follow $a_{ij} \sim \mathcal{N}(0, 0.2^2)(i \neq j)$ and $a_{ii} = -1$.
