# Supplementary material for: An ecological framework to understand the efficacy of fecal microbiota transplantation
Source: Nat Commun. 2020 Jul 3;11:3329. doi: 10.1038/s41467-020-17180-x (PMC7334230; doi:10.1038/s41467-020-17180-x)
Supplement: Supplementary file 3 — Reporting Summary [file 41467_2020_17180_MOESM3_ESM.pdf]

nature research

Corresponding author(s): Yang-Yu Liu

Last updated by author(s): May 31, 2020

Reporting Summary

Nature Research wishes to improve the reproducibility of the work that we publish. This form provides structure for consistency and transparency in reporting. For further information on Nature Research policies, see Authors & Referees and the Editorial Policy Checklist .

Statistics

For all statistical analyses, confirm that the following items are present in the figure legend, table legend, main text, or Methods section.

n/a	Confirmed	
☐	☒	The exact sample size (*n*) for each experimental group/condition, given as a discrete number and unit of measurement
☐	☒	A statement on whether measurements were taken from distinct samples or whether the same sample was measured repeatedly
☐	☒	The statistical test(s) used AND whether they are one- or two-sided<br>*Only common tests should be described solely by name; describe more complex techniques in the Methods section.*
☒	☐	A description of all covariates tested
☒	☐	A description of any assumptions or corrections, such as tests of normality and adjustment for multiple comparisons
☐	☒	A full description of the statistical parameters including central tendency (e.g. means) or other basic estimates (e.g. regression coefficient) AND variation (e.g. standard deviation) or associated estimates of uncertainty (e.g. confidence intervals)
☐	☒	For null hypothesis testing, the test statistic (e.g. *F*, *t*, *r*) with confidence intervals, effect sizes, degrees of freedom and *P* value noted<br>*Give P values as exact values whenever suitable.*
☒	☐	For Bayesian analysis, information on the choice of priors and Markov chain Monte Carlo settings
☒	☐	For hierarchical and complex designs, identification of the appropriate level for tests and full reporting of outcomes
☒	☐	Estimates of effect sizes (e.g. Cohen's *d*, Pearson's *r*), indicating how they were calculated

Our web collection on statistics for biologists contains articles on many of the points above.

Software and code

Policy information about availability of computer code

Data collection	No software was used for data collection.
Data analysis	Matlab-R2018a and R-3.5.2 code used in this work are available at https://github.com/xiaoyandong08/FMT_simulation_framework.

For manuscripts utilizing custom algorithms or software that are central to the research but not yet described in published literature, software must be made available to editors/reviewers. We strongly encourage code deposition in a community repository (e.g. GitHub). See the Nature Research guidelines for submitting code & software for further information.

Data

Policy information about availability of data

All manuscripts must include a data availability statement. This statement should provide the following information, where applicable:

- Accession codes, unique identifiers, or web links for publicly available datasets
- A list of figures that have associated raw data
- A description of any restrictions on data availability

Data analyzed in this work are available at https://github.com/xiaoyandong08/FMT_simulation_framework.

Field-specific reporting

Please select the one below that is the best fit for your research. If you are not sure, read the appropriate sections before making your selection.

☐ Life sciences ☐ Behavioural & social sciences ☒ Ecological, evolutionary & environmental sciences

For a reference copy of the document with all sections, see nature.com/documents/nr-reporting-summary-flat.pdf

Ecological, evolutionary & environmental sciences study design

All studies must disclose on these points even when the disclosure is negative.

Study description	We proposed an ecological modeling framework to simulate the FMT process. Besides the simulation results, we analyzed both in vitro and in vivo microbiome datasets to test our theoretical predictions.
Research sample	An interaction matrix inferred from the mouse experiments of antibiotic-mediated CDI was retrieved from the paper entitled "Ecological modeling from time-series inference: insight into dynamics and stability of intestinal microbiota".<br>An ecological network involving the so-called GnotoComplex microflora (a mixture of human commensal bacterial type strains) and C. difficile was retrieved from the paper entitled "MDSINE: Microbial Dynamical Systems INference Engine for microbiome time-series analyses".<br>A real FMT data from a clinical trial was requested from the corresponding author of a paper entitled "Predicting recurrence of Clostridium difficile infection following encapsulated fecal microbiota transplantation".
Sampling strategy	No sample size calculation was performed.
Data collection	1. The synthetic data were generated by population model and the parameters are described in figure captions and Supplementary Note 1.<br>2. An interaction matrix inferred from the mouse experiments of antibiotic-mediated CDI was retrieved from the paper entitled "Ecological modeling from time-series inference: insight into dynamics and stability of intestinal microbiota".<br>3. An ecological network involving the so-called GnotoComplex microflora (a mixture of human commensal bacterial type strains) and C. difficile was retrieved from the paper entitled "MDSINE: Microbial Dynamical Systems INference Engine for microbiome time-series analyses".<br>4. The clinical FMT dataset was requested from the corresponding author of a paper entitled "Predicting recurrence of Clostridium difficile infection following encapsulated fecal microbiota transplantation".<br>All the data were collected by the first author.
Timing and spatial scale	N/A
Data exclusions	N/A
Reproducibility	Reproducibility of our synthetic data and simulation results is ensured by performing internal technical review. Reproducibility of published datasets (that were re-analyzed in our work) can be found from the following original papers:<br>1. Ecological modeling from time-series inference: insight into dynamics and stability of intestinal microbiota<br>2. MDSINE: Microbial Dynamical Systems INference Engine for microbiome time-series analyses<br>3. Predicting recurrence of Clostridium difficile infection following encapsulated fecal microbiota transplantation
Randomization	N/A
Blinding	N/A

Did the study involve field work? ☐ Yes ☒ No

Reporting for specific materials, systems and methods

We require information from authors about some types of materials, experimental systems and methods used in many studies. Here, indicate whether each material, system or method listed is relevant to your study. If you are not sure if a list item applies to your research, read the appropriate section before selecting a response.

Materials & experimental systems

n/a	Involved in the study	
☒	☐	Antibodies
☒	☐	Eukaryotic cell lines
☒	☐	Palaeontology
☒	☐	Animals and other organisms
☐	☒	Human research participants
☐	☒	Clinical data

Methods

n/a	Involved in the study	
☒	☐	ChIP-seq
☒	☐	Flow cytometry
☒	☐	MRI-based neuroimaging

Human research participants

Policy information about studies involving human research participants

Population characteristics	The FMT clinical trial data was kindly shared by the corresponding author of the paper entitled "Predicting recurrence of Clostridium difficile infection following encapsulated fecal microbiota transplantation". We just analyzed the de-identified OTU table to validate our theoretical predictions. The population characteristics can be found in the original paper.
Recruitment	N/A
Ethics oversight	N/A

Note that full information on the approval of the study protocol must also be provided in the manuscript.

Clinical data

Policy information about clinical studies

All manuscripts should comply with the ICMJEguidelines for publication of clinical research and a completedCONSORT checklist must be included with all submissions.

Clinical trial registration	The FMT clinical trial data was kindly shared by the corresponding author of the paper entitled "Predicting recurrence of Clostridium difficile infection following encapsulated fecal microbiota transplantation". We just analyzed the de-identified OTU table to validate our theoretical predictions. The clinical trial registration can be found in the original paper.
Study protocol	N/A
Data collection	N/A
Outcomes	N/A
